# Supplementary material for: Complexome profiling of the Chlamydomonas psb28 mutant reveals TEF5 as an early PSII assembly factor
Source: Plant Cell. 2025 Mar 17;37(6):koaf055. doi: 10.1093/plcell/koaf055 (PMC12164590; doi:10.1093/plcell/koaf055)
Supplement: koaf055_Supplementary_Data [file koaf055_supplementary_data.zip › psb28_interactive.zip!/plots/intensity/qualityPlots/peptides/rpl5_Cre-1.g2717059.t1.p1.html]

### Cre-1.g2717059.t1.p1

Trivial name: rpl5  
SeqCoverage [median]: 11.70  
Mass: 20.24 kDa  
  
 

| Sequence | MissedCleavage | Mass [Da] | wtIntensity | muIntensity | Score |
| --- | --- | --- | --- | --- | --- |
| GIGDASQNQK | 0 | 1016.49 | 261.88 | 318.37 | 121.62 |
|  |  |  | 261.9 | 318.4 |  |
